# Analysis of Hollow Fiber Temperature Sensor Filled with Graphene-Ag Composite Nanowire and Liquid

**DOI:** 10.3390/s16101656

**Published:** 2016-10-08

**Authors:** Wei Xu, Jianquan Yao, Xianchao Yang, Jia Shi, Junfa Zhao, Cheng Zhang

**Affiliations:** 1 College of Precision Instrument and Opto-Electronics Engineering, Tianjin University, Tianjin 300072, China; xuwei@tjpu.edu.cn (W.X.); yangxianchao@tju.edu.cn (X.Y.); tjushijia@tju.edu.cn (J.S.); 2 School of Electronics and Information Engineering, Tianjin Polytechnic University, Tianjin 300387, China; johnfar@163.com (J.Z.); zhangcheng@tjpu.edu.cn (C.Z.)

**Keywords:** hollow fiber, birefringence, graphene-Ag composite nanowire, surface plasmon resonance (SPR)

## Abstract

A hollow fiber temperature sensor filled with graphene-Ag composite nanowire and liquid is presented and numerically characterized. The coupling properties and sensing performances are analyzed by finite element method (FEM) using both wavelength and amplitude interrogations. Due to the asymmetrical surface plasmon resonance sensing (SPR) region, the designed sensor exhibits strong birefringence, supporting two separate resonance peaks in orthogonal polarizations. Results show that *x*-polarized resonance peak can provide much better signal to noise ratio (SNR), wavelength and amplitude sensitivities than *y*-polarized, which is more suitable for tempertature detecting. The graphene-Ag composite nanowire filled into the hollow fiber core can not only solve the oxidation problem but also avoid the metal coating. A wide temperature range from 22 ${}^{\circ }$C to 47 ${}^{\circ }$C with steps of 5 ${}^{\circ }$C is calculated and the temperature sensitivities we obtained are 9.44 nm/${}^{\circ }$C for *x*-polarized and 5.33 nm/${}^{\circ }$C for *y*-polarized, much higher than other sensors of the same type.

## 1. Introduction

Photonics crystal fiber (PCF) [1,2], which is also called micro-structured optical fiber (MOF) has attracted considerable research interest in recent years due to its special structure and unique properties. PCFs are usually divided into two categories according to the guiding mechanisms. One category is refractive index-guiding, the other is photonic bandgap(BG)-guiding. Different kinds of PCFs can introduce some new applications by filling the air holes with different materials [3]. Moreover, it is possible to infiltrate functional materials into the air holes of those PCFs, which can tune the effective refractive index ($n_{eff}$) of the core guided mode efficiently, solving the mode coupling problem. Recently, various kinds of sensors based on PCF have been reported. They are used to measure factors such as temperature [4], strain [5], vibration [6], twist [7], refractive index (RI) [8], gas absorption [9], magnetic field [10] and so on, which are widely applied in chemical, physical and biochemical sensing fields.

Surface plasmon (SP) is a charge density wave of free electrons that occurs on the surface of a thin metal film interfacing with an adjacent dielectric. The amplitude of enhanced electric field reaches its maximum value at the metal surface and decays exponentially away from it [11]. Surface plasmon resonance (SPR) occurs at a certain frequency under the condition of phase matching between SPs and *p*-polarized incident light. Plasmon modes can also form on the metal wire [12] and the localized SPR (LSPR) [13] can be excited. Then, the metal coating or selective metal coating problem can be avoided by filling metal nanoparticles. Luan et al. [14] presented a temperature sensor based on PCF filled with silver nanowires and achieved a sensitivity 4 nm/${}^{\circ }$C. Peng et al. [3] proposed the same type sensor with PCF selectively filled with liquid and gold nanoparticles, and a temperature sensitivity of −5.5 nm/${}^{\circ }$C is realized by an experiment using the bandgap-like effect.

In this work, a temperature sensor based on a hollow fiber (HF) is presented and numerically analyzed. The air hole in the fiber core is filled with graphene-Ag composite nanowire and liquid (n=1.55 at 26.9 ${}^{\circ }$C, $\alpha =-4.15\times 10^{-4}/$${}^{\circ }$C) to form the sensing region. The RI of the liquid is higher than fused silica, which can satisfy the condition of total reflection. As the silver nanowire is asymmetrical to the fiber core, the sensor exhibits strong birefringence. One mode (*y-pol*) is polarized essentially parallel to the axis of symmetry and the other mode (*x-pol*) is orthogonal to it. The grapheme-Ag composite nanowire can not only solve the metal coating problem but also improve the sensitivity. Simulation results show that *x-pol* is better for temperature detecting due to its higher signal to noise ratio (SNR), wavelength and amplitude sensitivities than *y-pol*. A wide temperature range from 22 ${}^{\circ }$C to 47 ${}^{\circ }$C with steps of 5 ${}^{\circ }$C is calculated and the increased temperature sensitivities obtained are 9.44 nm/${}^{\circ }$C for *x-pol* and 5.33 nm/${}^{\circ }$C for *y-pol*, which are much higher than those in other sensors of same type as described in [3,14].

## 2. Sensor Design and Numerical Modeling

**Figure 1:**
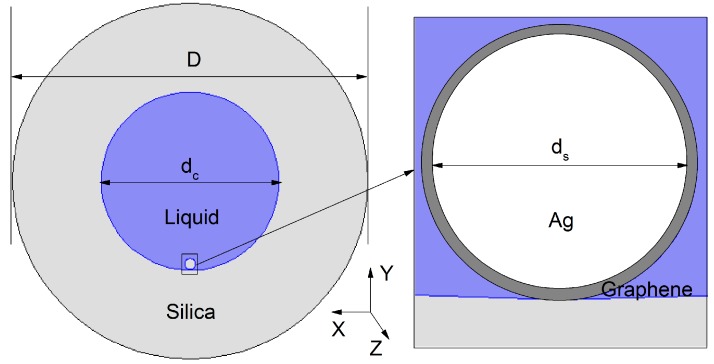
Cross section of the designed temperature sensor.

The cross section of the designed sensor is shown in Figure 1. The diameters of the fiber, the core, and the nanowire are *D* = 40 μm, $d_{c}$ = 20 μm, and $d_{s}$ = 1 μm, respectively. The nanowire and the liquid are filled into the fiber core and eventually the nanowire sinks to the bottom of the inner surface because of gravity effects. The filling process should be easy to operate because the holes of the HFs are available over a relatively wide range of diameter, from several hundred microns to several millimeters or more [8,15]. Graphene’s good features as high electron density of hexagonal rings, high surface to volume ratio, broadband optical and plasmonic properties make it an appropriate candidate to be used as a functional coating material for existing plasmonic devices. The graphene-Ag composite nanowire can solve the metal coating and silver oxidation problem, and improve the sensitivity [16,17,18]. The RI of graphene is calculated by [17]:

(1) $$n_{g}=3+\frac{iC_{1}\lambda }{3}\;,$$

where $C_{1}$ = 5.446 μm${}^{-1}$ , $n_{g}$ is the RI of graphene and *λ* is the vacuum wavelength. The total thickness of the graphene layer is 0.34 × *L* as each single layer is 0.34 nm, where *L* is the number of graphene layers. In Figure 1, the Ag nanowire is coated with a single graphene layer. The thermo-optic coefficient of the filling liquid we used is $\alpha =-4.15\times 10^{-4}/$${}^{\circ }$C as described in [19]. The background material of the HF is fused silica and the RI is assumed to be 1.45. The RI of Ag is referred to in [20].

We use the finite element method (FEM) to calculate with COMSOL Multiphysics software. The boundary condition is the perfectly matched layer (PML) and the whole section of the sensor in Figure 1 is divided into 183,578 triangular subdomains. The modal analysis is simulated in the XY plane and the light is propagated along the *Z* direction.

**Figure 2:**
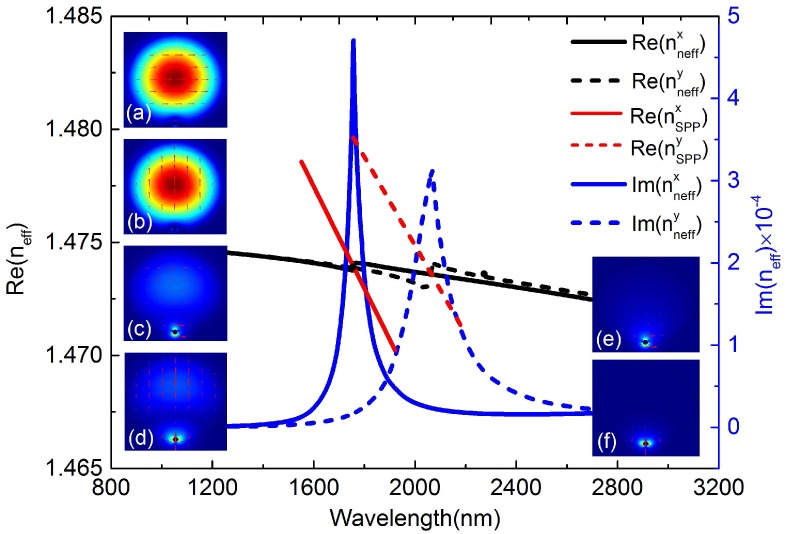
(Left) Dispersion relations of the core guided modes and the plasmon modes at temperature *T* = 22 ${}^{\circ }$C. (Insets) Electric field distributions of (**a**) core guided mode of *x-pol* at *λ* = 1.4 μm; (**b**) core guided mode of *y-pol* at *λ* = 1.4 μm; (**c**) core guided mode of *x-pol* at *λ* = 1.757 μm (resonance wavelength); (**d**) core guided mode of *y-pol* at *λ* = 2.068 μm (resonance wavelength); (**e**) plasmon mode of *x-pol* at *λ* = 1.75 μm; (**f**) plasmon mode of *y-pol* at *λ* = 2.06 μm.

The dispersion relationship between core guided modes and plasmon modes at temperature *T* = 22 ${}^{\circ }$C is shown in Figure 2. Obviously, we can see that the asymmetrical SPR region leads to strong birefringence and most of the light is coupled into a particular direction. Two real parts of $n_{eff}$ of the core guided modes (black solid and black dotted curves) and plasmon modes (red solid and red dotted curves) result in two different intersections and resonance peaks (blue solid and blue dotted curves) for the same analyte of RI. When SPR occurs, most of the energy transfers from the core-guided mode to the plasmon mode and the resonant loss peak will be formed at the resonance wavelength.

## 3. Results and Discussion

**Figure 3:**
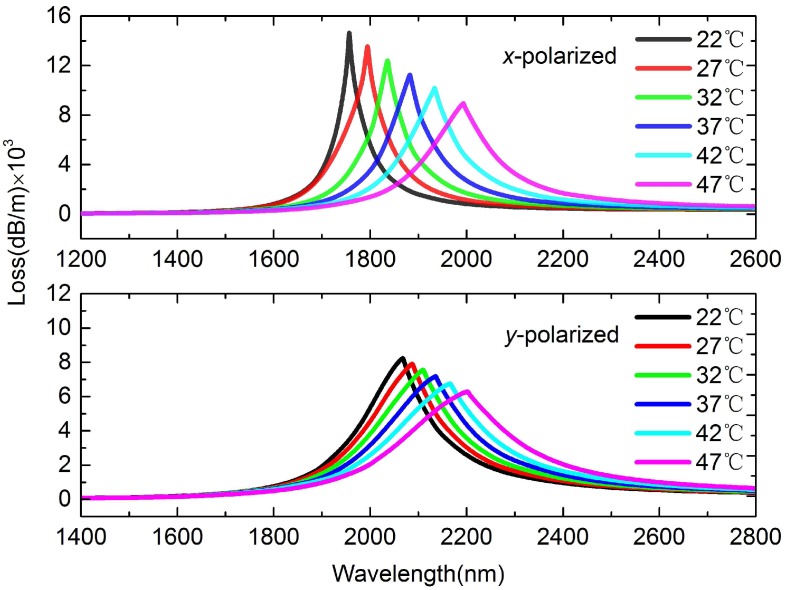
Loss spectra of *x*- and *y-pol* when *T* changes from 22 ${}^{\circ }$C to 47 ${}^{\circ }$C with steps of 5 ${}^{\circ }$C.

To investigate the sensor’s performance, the temperature at *T* = 22 ${}^{\circ }$C, 27 ${}^{\circ }$C, 32 ${}^{\circ }$C, 37 ${}^{\circ }$C, 42 ${}^{\circ }$C, 47 ${}^{\circ }$C is illustrated in Figure 3. Clearly, we can see that the peak intensities of *x-pol* are about $6\times 10^{3}$ dB higher than *y-pol*. Moreover, the resonance wavelength range of *x-pol* is 1750 nm∼2000 nm, shorter than *y-pol* of 2060 nm∼2200 nm due to the strong birefringence. In contrast with the blue-shift of traditional SPR temperature sensors, the resonance wavelength in our designed sensor changes to a longer wavelength and the intensity of peak loss gradually decreases. The differences are mainly caused by the different ways of liquid filling. In traditional SPR temperature sensors, the liquid with large thermo-optic coefficient is injected into the air holes located at the fiber cladding. Then the $n_{eff}$ of the plasmon mode will decrease when temperature increases. It approaches the RI of the liquid, resulting in the phase matching point (see Figure 2) with core guided mode shifts to the shorter wavelength. However, in this sensor, the liquid and nanowire are filled into the fiber core, thus the $n_{eff}$ of core guided mode approaches the RI of the liquid. Then, when temperature increases, the $n_{eff}$ of core guided mode will decrease, resulting in the phase matching point with plasmon mode corresponding to the resonance wavelength (see Figure 2) shifts to the longer wavelength.

In wavelength interrogation, changes in temperature can be detected though measuring the shift of resonance peak. The wavelength sensitivity is defined as [14]:

(2) $$S_{\lambda }\left(nm{/}^{\circ }C\right)=\frac{\partial \lambda_{peak}}{\partial n_{a}}\;,$$

According to Equation (2), when the temperature changes from 22 ${}^{\circ }$C to 27 ${}^{\circ }$C, the wavelength sensitivities of *x*- and *y-pol* are 7.8 nm/${}^{\circ }$C and 3.8 nm/${}^{\circ }$C, respectively.

The detection accuracy is closely related to the peak width. The narrower the width, the higher the detection accuracy. SNR is an important parameter to evaluate the peak width and can be calculated by [21]:

(3) $$SNR=\frac{\delta \lambda_{res}}{\delta \lambda_{0.5}}\;,$$

where $\delta \lambda_{0.5}$ is the peak width corresponding to 50% of peak loss and $\delta \lambda_{res}$ is the resonance wavelength shifts. According to Equation (3), the SNRs of *x*- and *y-pol* are 0.75 and 0.12, respectively.

Another method that is frequently-used is the amplitude interrogation. Suppose that the light wavelength is *λ*, the analyte RI is $n_{a}$, the HF length is *L*, the function of core guided mode loss and wavelength is $\alpha (\lambda ,n_{a})$. The amplitude sensitivity is defined as [22]:

(4) $$S\left(RIU^{-1}\right)=\frac{1}{\alpha (\lambda ,n_{a})}\frac{\partial \alpha (\lambda ,n_{a})}{\partial n_{a}}\;,$$

**Figure 4:**
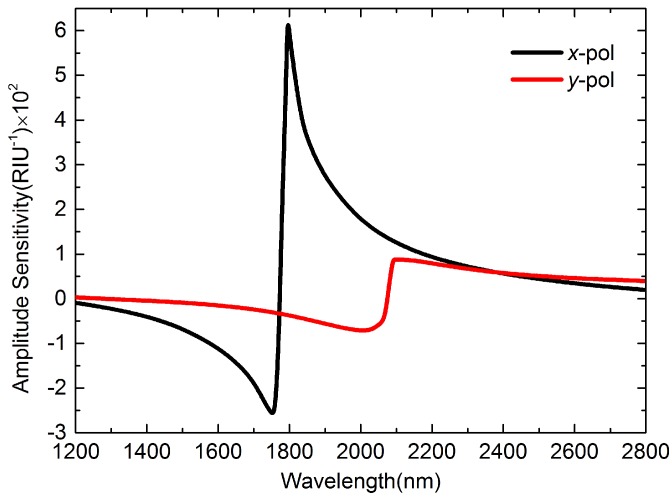
Amplitude sensitivities of *x*- and *y-pol* peaks when *T* changes form 22 ${}^{\circ }$C to 27 ${}^{\circ }$C.

Figure 4 depicts the amplitude sensitivities of *x*- and *y-pol* when temperature changes from 22 °C to 27 ${}^{\circ }$C. From Figure 4 we know that the maximum *S* of *x-pol* is 644.5 RIU${}^{-1}$, much higher than 87.9 RIU${}^{-1}$ of *y-pol*.

**Figure 5:**
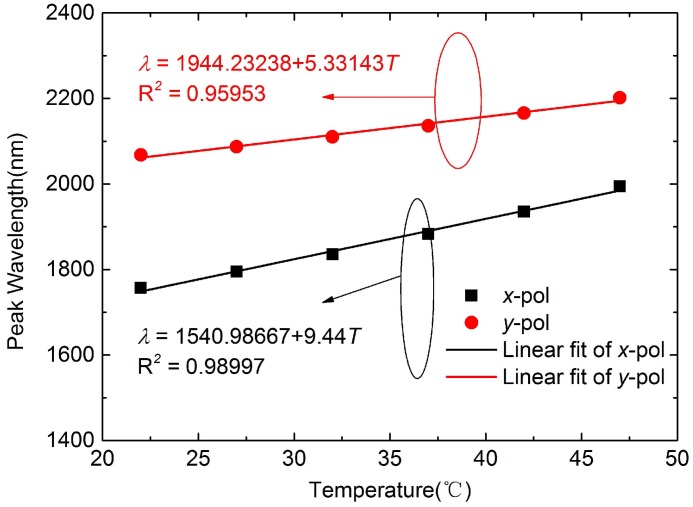
Relationships between resonance peaks and temperature.

The relationships between resonance peaks and temperature are described in Figure 5. The curves of matching function can be set up as *λ* = 1540.98667 + 9.44*T* for *x-pol* and *λ* = 1944.23238 + 5.33143*T* for *y-pol*. The correlations are respectively 0.98997 and 0.95953 between fitting and experimental results, showing a good linear relationship. Then the temperature sensitivities 9.44 nm/${}^{\circ }$C for *x-pol* and 5.33 nm/${}^{\circ }$C for *y-pol* can be obtained,which are higher than the 4 nm/${}^{\circ }$C of Ag nanowire filled temperature sensor [14].

## 4. Conclusions

In this paper, we present and numerically characterize a hollow fiber temperature sensor filled with graphene-Ag composite nanowire and liquid. The coupling properties and sensing performances are analyzed by FEM using wavelength and amplitude interrogations. The sensor exhibits strong birefringence because of the asymmetrically SPR sensing region and simulation results show that *x-pol* is better suited to temperature detecting in consideration of SNR, wavelength and amplitude sensitivities. A wide temperature range from 22 ${}^{\circ }$C to 47 ${}^{\circ }$C with steps of 5 ${}^{\circ }$C is calculated and the high temperature sensitivities we obtained are 9.44 nm/${}^{\circ }$C for *x-pol* and 5.33 nm/${}^{\circ }$C for *y-pol*. This work is a promising step in developing a highly sensitive, real-time, fast response and distributed SPR temperature sensor.
